# Increased Serum Interleukin-2 Levels Are Associated with Abnormal Peripheral Blood Natural Killer Cell Levels in Patients with Active Rheumatoid Arthritis

**DOI:** 10.1155/2020/6108342

**Published:** 2020-09-15

**Authors:** Baochen Li, Qiaoling Guo, Yanyan Wang, Rui Su, Chong Gao, Jinfang Zhao, Xiaofeng Li, Caihong Wang

**Affiliations:** ^1^Department of Rheumatology, The Second Hospital of Shanxi Medical University, Taiyuan, Shanxi 030001, China; ^2^Department of Pathology, Brigham and Women's Hospital, Harvard Medical School, Boston, MA, USA; ^3^Department of Medical Statistics, Shanxi Medical University, Taiyuan, Shanxi 030001, China

## Abstract

**Objective:**

To investigate the relationship between serum interleukin-2 (IL-2) levels and disease activity, absolute numbers of peripheral lymphocyte subsets, autoantibodies, and associated cytokines in patients with rheumatoid arthritis (RA).

**Methods:**

This study included 106 patients with RA, evaluated their disease activity (DAS28 score), and divided them into disease remission (DAS28 ≤ 2.6), low disease activity (DAS28 ≤ 3.2), and moderate-high disease activity (*DAS*28 > 3.2) groups. Flow cytometry was used to detect the absolute numbers of peripheral lymphocyte subpopulations and CD4+ T cell subsets in each group, and serum cytokine levels were measured using cytometric bead array.

**Results:**

Serum IL-2 levels in RA patients were positively correlated with disease activity and rheumatoid factor titers (*p* < 0.001 and *p* = 0.045, respectively), and multiple regression analysis revealed that serum IL-2 levels were an independent factor affecting disease activity. Serum IL-2 levels were positively correlated with Th17/Treg ratios (*p* = 0.013). Compared with the remission group, peripheral lymphocyte and CD4+ T lymphocyte subsets in patients with active RA decreased to varying degrees; however, the numbers of peripheral natural killer (NK) cells were significantly higher in the moderate-high disease activity group than in the remission (*p* = 0.046) and low disease activity (*p* = 0.020) groups; the percentages of NK cells had the same trend. In addition, the number and percentage of NK cells were positively correlated with serum IL-2 levels (*p* = 0.018 and *p* = 0.006, respectively).

**Conclusions:**

In RA patients, serum IL-2 levels were not only correlated with patients' disease activity and autoantibody levels but were also involved in their Th17/Treg immune imbalance. In addition, in patients with active RA, NK cell levels were abnormally elevated, possibly due to high serum levels of IL-2.

## 1. Introduction

Rheumatoid arthritis (RA) is a systemic, inflammatory, autoimmune disease that causes progressive joint damage that can lead to lifelong disability. This systemic disease is characterized by synovial inflammatory cell infiltration, synovial hyperplasia, angiogenesis, cartilage, and bone erosion [1]. The cytokine-mediated pathway is central to the pathogenesis of RA. At the affected joint, T cells and other infiltrating immune cells associated with the disease release a variety of cytokines, which are important mediators of cell differentiation, inflammation, immune pathology, and immune response [2]. Among them, interleukin 2 (IL-2) was once considered an important proinflammatory factor because it not only promotes the expansion of T cells [3] and enhances the ability of natural killer (NK) cells [4] but also strengthens the body's antitumor immune response [5]. However, recent studies have demonstrated the pleiotropy of IL-2: it both activates traditional T cells to promote the immune response and promotes regulatory T (Treg) cell proliferation to control the inflammatory response and maintain immune tolerance as a cytokine necessary for Treg cell development and homeostasis [6, 7]. Moreover, low-dose IL-2 has been used to treat systemic lupus erythematosus (SLE), type 1 diabetes (T1D), and other autoimmune diseases and has shown some efficacy [8, 9]. However, in RA patients, the relationships among serum IL-2 levels and disease activity, Th17/Treg immune balance, peripheral blood lymphocyte subpopulation counts, RA-specific autoantibodies such as rheumatoid factor (RF) and anticyclic citrullinated peptide (CCP), and serum levels of other cytokines have rarely been reported.

Therefore, with the advancement of detection technology and clinical application of the absolute numbers of lymphocyte subpopulations, the purpose of this study was to investigate the relationship between serum IL-2 levels and various clinical indicators, lymphocytes, and other cytokines in RA patients to clarify the relationship between serum IL-2 levels and RA.

## 2. Materials and Methods

### 2.1. Clinical Data

In total, 106 RA patients, comprising 26 males and 80 females with an average age of 46.96 ± 15.10 years, were recruited from the Rheumatology Department of the Second Hospital of Shanxi Medical University (Taiyuan, China) between March and October 2018. All patients were diagnosed according to the revised American College of Rheumatology/European League against Rheumatism classification criteria for RA of 2010 [10]. Exclusion criteria were other autoimmune diseases, severe infections, tumors, hypertension, diabetes, other chronic diseases, and receiving low-dose human recombinant IL-2 treatment. The retrospectively collected clinical and serological parameters of these patients included white blood cell, hemoglobin, lymphocyte, and platelet counts; erythrocyte sedimentation rate (ESR); C-reactive protein (CRP), RF, anti-CCP antibody (*α*-CCP), and cytokine levels; absolute numbers of peripheral lymphocytes; and T cell subset data. All blood samples required for clinical indicators were collected after fasting on the morning of the patient's consultation.

Based on the patient's clinical symptoms and laboratory tests, a disease activity score (DAS28) was evaluated: DAS28 is scored on 28 joints including 10 metacarpophalangeal joints of both hands, 10 proximal interphalangeal joints of both hands, and both wrist joints, both elbow joints, both shoulder joints, and both knee joints. The DAS28 score is calculated as follows: tender joint count (TJC), swollen joint count (SJC), and ESR, DAS28 = (0.56 × TJC + 0.28 × SJC + 0.7 × ln ESR) × 1.08 + 0.16. Based on the calculated values, the patients were grouped into the disease remission (DAS28 ≤ 2.6), low disease activity (*DAS*28 ≤ 3.2), and moderate-high disease activity (*DAS*28 > 3.2) groups. The differences in age and sex among the three groups were not statistically significant (*p* > 0.1). This study was approved by the Ethics Committee of the Second Hospital of Shanxi Medical University (Approval (2019) KY No. (105)), and all subjects provided a written informed consent.

### 2.2. Flow Cytometry for Absolute Number of Peripheral Lymphocytes and T Cell Subsets

To determine the percentage and absolute numbers of peripheral lymphocytes and CD4+ T cell subsets, peripheral blood samples (2 ml) were collected from each patient in EDTA anticoagulant tubes according to the manufacturer's recommendations (BD Biosciences, Franklin Lakes, NJ, USA).

#### 2.2.1. Absolute Numbers of Peripheral Blood Lymphocytes

**Figure 1:**
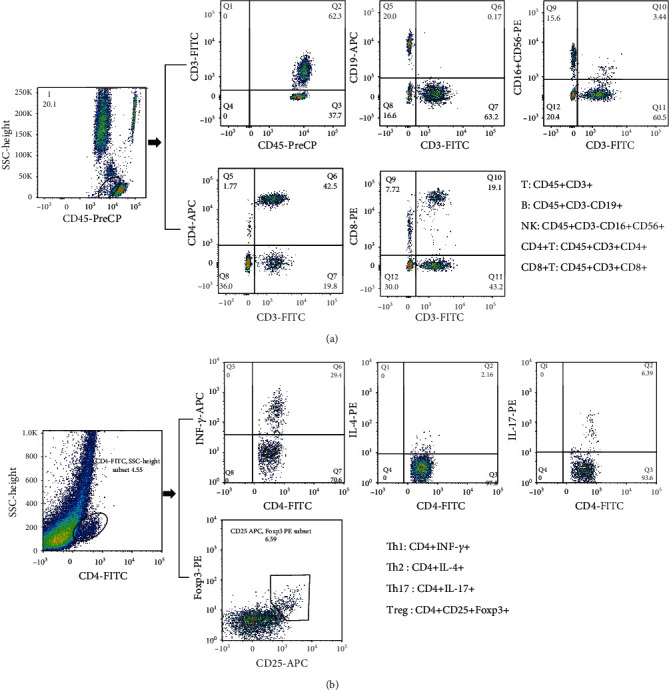
Phenotypic characterization of lymphocyte subpopulations by flow cytometry. (a) Representative flow cytometry analysis of peripheral lymphocytes. T: CD45+ CD3+; B: CD45+ CD3- CD19+; NK: CD45+ CD3- CD16+ CD56+; CD4+ T: CD45+ CD3+ CD4+; CD8+ T: CD45+ CD3+ CD8+. (b) Representative flow cytometry analysis of CD4+ T cell subsets. All dot plot analysis is of CD4+ gated lymphocyte. Th1:CD4+ INF-*γ*+; Th2: CD4+ IL-4+; Th17: CD4+ IL-17+; Treg: CD4+ CD25+ Foxp3+.

Two BD Trucount tubes containing a known number of fluorescent beads were serially numbered (A and B), and 50 *μ*l blood samples were added to the tubes by reverse loading. Then, 20 *μ*l of anti-CD3-FITC/CD-8PE/CD45PercP/CD4APC antibodies was added to tube A, and 20 *μ*l of CD3FITC/CD16+56-PE/CD45-PercP/CD19-APC antibodies was added to tube B (all from BD Biosciences). The contents of the tubes were mixed and incubated at room temperature for 15–20 min in the dark. Then, 450 *μ*l XFACS hemolysin was added and mixed thoroughly, followed by incubation at room temperature for 15 min in the dark. In total, 15000 cells were obtained for detection in 24 h and analyzed by flow cytometry and MultiSET software (BD Biosciences) (Figure 1(a)).

#### 2.2.2. CD4+ T Lymphocyte Subset Detection

*(1) Th1, Th2, and Th17 Cell Cultures and Labeling*. An 80 *μ*l blood sample together with 10 *μ*l phorbol myristate acetate working solution (final concentration, 30 ng/ml), 10 *μ*l ionomycin working solution (final concentration, 750 ng/ml), and 1 *μ*l GolgiStop were incubated at 37°C, 5% CO_2_ for 5 h. Then, the samples were divided into two tubes, followed by staining with anti-CD4-FITC antibodies at room temperature in the dark for 30 min. The tubes were mixed with 1 ml freshly prepared fixation/permeabilization solution and placed in an incubator at 4°C in the dark for 30 min. Anti-IL-4-PE and anti-interferon gamma (IFN-*γ*)-APC were added to the A tube; anti-FITC-CD4 and anti-IFN-*γ*-APC (intracellular staining) were used to detect Th1 cells and anti-FITC-CD4, and anti-IL-4-PE (intracellular staining) were used to detect Th2 cells. Anti-human IL-17-PE (intracellular staining) was added to the B tube for Th17 cell analysis. The two tubes of cells were stored at room temperature for 30 min in the dark and washed with phosphate-buffered saline (PBS). The absolute numbers of CD4+ T lymphocyte subsets were automatically detected using BD Multitest software (BD Biosciences). All immunofluorescence antibodies were purchased from BD Biosciences (Figure 1(b)).

*(2) Detection of Treg Cells*. Anti-CD4-FITC and anti-CD25-APC were added to an 80 *μ*l blood sample and incubated at room temperature in the dark for 30 min. Then, 1 ml freshly prepared fixation/permeabilization solution was added to each tube, mixed, and incubated at 4°C for 30 min. Anti-FOXP3-PE (intracellular staining) was added and incubated at room temperature for 30 min in the dark, followed by washing with PBS and detection of Treg cells using flow cytometry. All immunofluorescence antibodies were purchased from BD Biosciences (Figure 1(b)).

*(3) Flow Cytometry*. The stained cells were measured using flow cytometry (Calibur; BD Biosciences) within 24 h. Based on the scatter plot of the forward angular scattered light relative to the lateral angular dispersive light (side scatter (SSC)), the lymphocytes were gated to distinguish lymphocytes, and CD4 was used to distinguish CD4+ T cells from the SSC gate; 10000 cells in the gate were taken. The relative percentages were obtained and analyzed using CellQuest software. The absolute number of cells in each subgroup was calculated as: cell absolute number = percentage of positive cells in each subset × absolute number of CD4 + T cells (cells/*μ*l) cells/*μ*l whole blood.

### 2.3. Detection of Cytokine Levels by Cytometric Bead Array

The serum was separated from 4 ml venous blood after 1–2 h and stored at -20°C. IL-2, IL-4, IL-6, IL-10, IFN-*γ*, and tumor necrosis factor-*α* (TNF-*α*) were measured using flow cytometry. A cytometric bead array (CBA) kit was purchased from Jiangsu Sage Biotechnology Co. Ltd. (Jiangsu, China) and used according to the manufacturer's instructions; the results were expressed as pg/ml.

### 2.4. Statistical Processing

The count data were measured using the chi-square goodness-of-fit test. The measurement data were tested using the Kolmogorov-Smirnov test and Levene's *t*-test; the mean ± standard deviation was used to represent the normality and homogeneity of variance. The independent sample *t*-test was used for comparisons between two groups; the analysis of variance method was used for comparisons among groups. Nonnormal distribution data were expressed as the median (*M*) and interquartile range and tested using the Kruskal-Wallis *H* test. Correlation analysis was performed using the Pearson or Spearman correlation. Difference was considered statistically significant at *p* ≤ 0.05. All statistical analyses were performed using SPSS software (ver. 23.0; SPSS Inc., Chicago, IL, USA).

## 3. Results

### 3.1. Differences in Serum IL-2 Levels in RA Patients in the Different Disease Activity Groups

**Figure 2:**
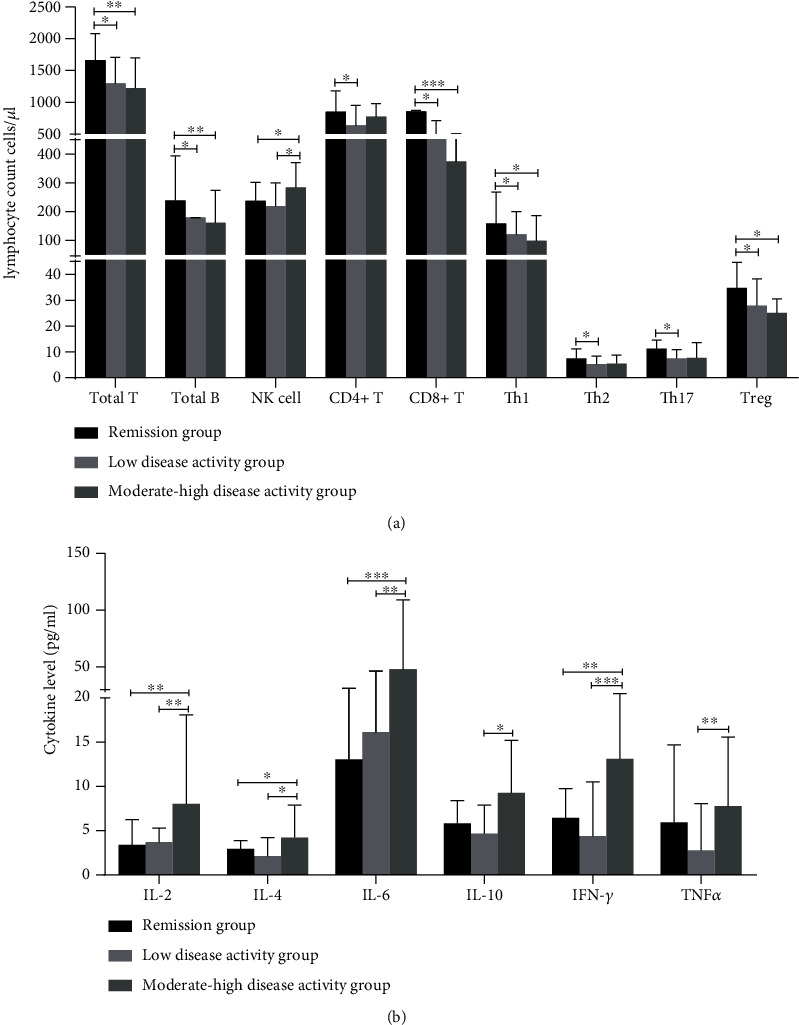
(a) Comparison of absolute lymphocyte and CD4+ T cell subset counts among the remission group (*n* = 33), low disease activity group (*n* = 38), and moderate-high disease activity group (*n* = 35). (b) Comparison of serum cytokine levels among the remission group (*n* = 33), low disease activity group (*n* = 35), and moderate-high disease activity group (*n* = 38) (^∗^*p* < 0.05, ^∗∗^*p* < 0.01, and ^∗∗∗^*p* < 0.001).

Among the 106 patients with RA, there was no statistically significant difference in the baseline clinical characteristics of the disease activity groups in terms of sex, age, height, or weight. In addition, there was no significant difference in the duration of prednisone use, TNF-*α* inhibitor treatment, or use of other medication (*p* > 0.05). However, prednisone dosage was significantly higher in the moderate-high disease activity group than in the disease remission group (*p* = 0.015) (Tables 1(a) and 1(b)). To explore the relationship between serum IL-2 levels and RA, we compared serum IL-2 levels among the groups and found that levels were significantly higher in the moderate-high disease activity group than in the remission and low disease activity groups (*p* = 0.002 and *p* = 0.001, respectively). However, there was no significant difference between the remission group and the low disease activity group (*p* = 0.893) (Table 2 and Figure 2(b)).

### 3.2. Relationships among Serum IL-2 or Other Inflammatory Cytokine Levels and Disease Activity in Patients with RA

**Figure 3:**
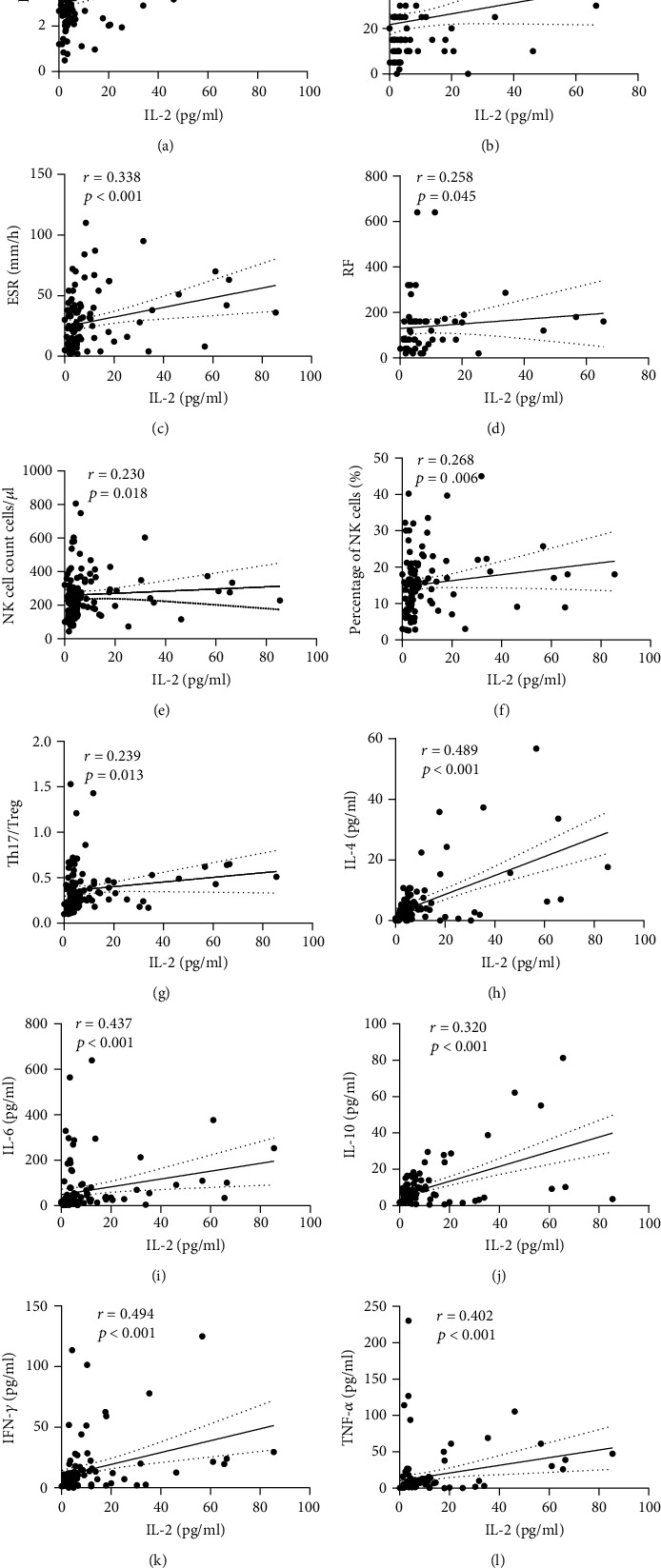
Correlation analysis between serum IL-2 level of RA patients and various clinical indicators. (a–c) Correlation analysis between serum IL-2 level of RA patients and DAS28, ESR, and VAS. (d) Correlation analysis between serum IL-2 level of RA patients and serum RF level (only 61 patients with RA had autoantibodies test results). (e, f) Correlation analysis between serum IL-2 level of RA patients and NK cell number and percentage of NK cells. (g) Correlation analysis between serum IL-2 level of RA patients and Th17/Treg ratio. (h–l) Correlation analysis between serum IL-2 level of RA patients and IL-4, IL-6, IL-10, INF-*γ*, and TNF-*α*. Shown are linear regression lines with interpolated 95% confidence interval curves (broken lines).

**Figure 4:**
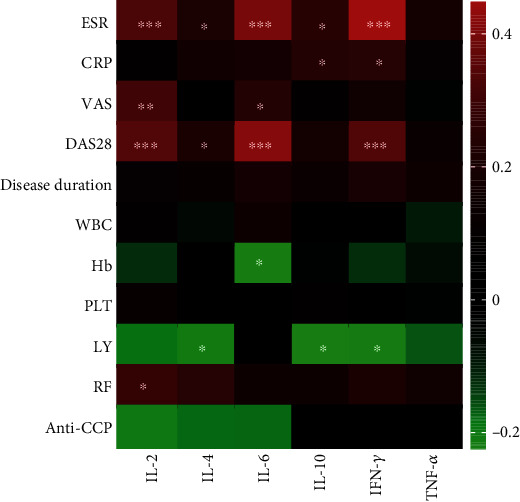
Heatmap of correlation of the serum cytokine levels of IL-2, IL-4, IL-6, IL-10, INF-*γ*, TNF-*α* with ESR, CRP, VAS, DAS28, disease duration, WBC, Hb, PLT, LY, RF, and anti-CCP in RA patients. (^∗^*p* < 0.05, ^∗∗^*p* < 0.01, and ^∗∗∗^*p* < 0.001 by Spearman's correlation test).

DAS28 scores were used to reflect the disease activity of RA patients. Serum IL-2 levels in RA patients were positively correlated with DAS28 scores (*r* = 0.339, *p* < 0.001), and with visual analogue scores (VASs) (*r* = 0.315, *p* = 0.003) that reflected patients' pain levels. The ESR is an inflammatory indicator that can also reflect the disease activity of RA patients to some extent. Our study found that serum IL-2 levels in RA patients were positively correlated with ESRs (*r* = 0.338, *p* < 0.001; Figures 3(a)–3(c)). In addition to serum IL-2 levels, serum levels of IL-4, IL-6, and INF-*γ* were also significantly positively correlated with DAS28 scores (Figure 4). To exclude the effects of other inflammatory cytokines, multiple linear regression was further applied to construct a regression model (*F* = 4.981, *p* = 0.001). The equation had statistical significance, and regression analysis showed that in RA patients; DAS28 scores were independently positively correlated with IL-2 levels (*β* = 0.237, *p* = 0.042) and IL-6 levels (*β* = 0.196, *p* = 0.044), *R*^2^ = 0.166 (Table 3). In addition, serum IL-2 levels in RA patients were significantly correlated with levels of other inflammatory cytokines including IL-4 (*r* = 0.489, *p* < 0.001), IL-6 (*r* = 0.437, *p* < 0.001), IL-10 (*r* = 0.320, *p* < 0.001), INF-*γ* (*r* = 0.494, *p* < 0.001), and TNF-*α* (*r* = 0.402, *p* < 0.001) (Figures 3(h)–3(l)).

### 3.3. Comparison of B Cells and Specific Antibodies among RA Patients in the Different Disease Activity Groups and Their Relationship with Serum IL-2 Levels

**Figure 5:**
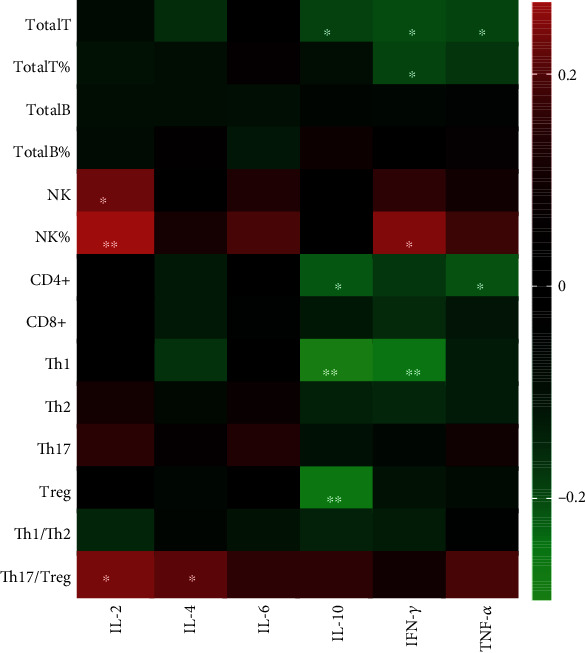
Heatmap of correlation of the serum cytokine levels of IL-2, IL-4, IL-6, IL-10, INF-*γ*, TNF-*α* with Total T, Total T%, Total B, Total B%, NK, NK%, CD4+, CD8+, Th1, Th2, Th17, Treg, Th1/Th2, and Th17/Treg (^∗^*p* < 0.05, ^∗∗^*p* < 0.01, and ^∗∗∗^*p* < 0.001 by Spearman's correlation test).

B lymphocytes and the specific antibodies they produce are of great significance for the diagnosis, classification, and efficacy of RA treatment. This study found that the numbers of peripheral B cells were significantly higher in the disease remission group than in the low disease activity and moderate-high disease activity groups (*p* = 0.029 and *p* = 0.001, respectively) (Table 4(a) and Figure 2(a)). There was no significant correlation between numbers of peripheral B cells and IL-2 levels (*p* > 0.05; Figure 5); however, we found a significant positive correlation between serum IL-2 levels and serum RF titers (*r* = 0.258, *p* = 0.045) but no significant correlation with serum anti-CCP antibody titers (*r* = −0.146, *p* = 0.271) (Figures 3(d) and 4).

### 3.4. Comparison of Th17/Treg Immune Balance and Its Relationship with Serum IL-2 Levels in RA Patients with Different Disease Activity Levels

The immune imbalance of Th17/Treg is an important pathogenic factor for RA. In this study, the Th17/Treg ratio was significantly higher in the moderate-high disease activity group than in the remission and low disease activity groups (*p* = 0.003 and *p* = 0.010, respectively) (Table 4(c)). In addition, the serum IL-2 levels of RA patients were positively correlated with Th17/Treg ratios in peripheral blood (*r* = 0.239, *p* = 0.013) (Table 5 and Figure 3(g)), while there was no significant correlation between Th17 and Treg cells in peripheral blood (*p* > 0.05; Table 5 and Figure 5). The numbers of peripheral Treg cells were significantly higher in the disease remission group than in the low disease activity and moderate-high disease activity groups (*p* = 0.005 and *p* < 0.001, respectively) (Table 4(c) and Figure 2(a)). Combined with the differences in IL-2 levels among the different disease activity groups, we found a parallel relationship between increased serum IL-2 levels and decreased peripheral Treg cells in patients with active RA. The remission group had significantly higher numbers of Th17 cells in peripheral blood compared to the low disease activity group (*p* = 0.012), whereas there was no significant difference among the other groups (*p* > 0.05; Table 4(c) and Figure 2(a)).

### 3.5. Comparison of NK Cell Levels in RA Patients in the Different Disease Activity Groups and the Relationship with Serum IL-2 Levels

NK cells may trigger osteoclast generation and bone destruction in RA [11]. This study found significantly higher numbers of peripheral NK cells in the moderate-high disease activity group than in the disease remission and low disease activity groups (*p* = 0.046 and *p* = 0.020, respectively) (Table 4(b) and Figure 2(a)). Moreover, the percentages of peripheral NK cells were significantly higher in the moderate-high disease activity group than in the remission and low disease activity groups (*p* < 0.001 and *p* = 0.001, respectively) (Table 4(b)). In addition, the numbers of NK cells in peripheral blood (*r* = 0.230, *p* = 0.018) and the percentages of NK cells (*r* = 0.268, *p* = 0.006) were positively correlated with serum IL-2 levels in RA patients (Table 5 and Figures 3(e) and 3(f)). Furthermore, the percentages of peripheral NK cells were significantly positively correlated with serum levels of INF-*γ* (*r* = 0.246, *p* = 0.011; Figure 5).

### 3.6. Differences in Other Lymphocyte Subsets among RA Patients in the Different Disease Activity Groups

With the exception of the abovementioned cells, the lymphocyte subsets of patients with active RA showed different degrees of decline compared with the remission group. The total numbers of T lymphocytes, CD8+ T lymphocytes, and Th1 cells were significantly lower in the low disease activity and moderate-high disease activity groups than in the remission group. The numbers of peripheral CD4+ T cells and Th2 cells were significantly lower in the low disease activity group than in the diseaseremission group. (Tables 4(b) and 4(c) and Figure 2(a)).

## 4. Discussion

Recent studies have shown that IL-2 is a key cytokine that affects the differentiation of immune cells and has pleiotropic effects: it not only activates effector T cells to promote immune response but also promotes Treg cell proliferation to maintain immune tolerance, playing important roles in infection, autoimmune diseases, and cancer. Therefore, in patients with RA, whether serum IL-2 levels are related to disease activity, whether they affect the development and differentiation of lymphocyte subsets and the potential therapeutic prospect of low-dose IL-2 in RA are clinical frontier hotspots.

This study mainly investigated RA patients in our hospital's Rheumatology Department; patients' serum IL-2 levels and the clinical significance thereof were evaluated by assessing patients' disease activity and detecting disease-related clinical indicators. Our results show that in RA patients, monitoring serum IL-2 levels is of great significance, because they are not only related to patients' disease activity and autoantibody levels but also participate in patients' Th17/Treg immune imbalance. Moreover, in patients with active RA, NK cells are abnormally elevated, and high levels of serum IL-2 may be an important predisposing factor.

### 4.1. Serum IL-2 Levels Are Associated with Disease Activity and Autoantibody Production in RA Patients

In RA patients, the DAS28 score, ESR, CRP, and other indicators can reflect the degree of systemic inflammation, disease activity, and therapeutic effect to a certain extent. In our study, immunological indicators were used to monitor disease activity. By studying the levels of cytokines and the numbers of lymphocyte subsets, disease activity was accurately evaluated; this is of important clinical significance in the selection of a treatment plan and the prognosis of patients. Our results show that IL-2 serum levels in RA patients were an independent factor affecting DAS28 scores and were significantly positively correlated with ESRs, indicating that serum IL-2 levels in these RA patients were correlated with inflammatory degree and disease activity.

In addition to chronic systemic inflammation, severe chronic arthralgia is a major concern for RA patients and seriously affects their daily life and mental health [12]. Although clinicians have traditionally considered pain a marker of inflammation, many studies have found that some inflammatory cytokines, including TNF-*α*, IL-1*β*, IL-6, and IL-17, can directly alter the response of pain-sensitive neurons to produce and maintain arthralgia [13] and depletion of these inflammatory cytokines can rapidly relieve patients' pain [14]. Although IL-2 does not directly mediate or aggravate the pain status of patients, in this study, RA patients' serum IL-2 levels were significantly positively correlated with their VAS pain scores and levels of pain-related cytokines such as IL-6 and TNF-*α*. In this study, elevated serum IL-2 levels in RA patients paralleled their levels of pain.

The production of autoantibodies is one of the characteristics of RA. It is generally accepted that RF and anti-CCP are indicators of RA severity, imaging progression, and joint damage [15]. Autoantibodies are derived from plasma cells derived from B cell differentiation; studies have shown that IL-2 mediates the conversion of initial B cells to plasma cells by activating the extracellular signal-related kinase/ETS domain-containing protein ELK1 signaling pathway [16], thereby promoting production of autoantibodies. Our study found that although serum IL-2 levels had no significant relationship with the numbers and percentages of peripheral B cells, they were positively correlated with serum RF titers, indicating that serum IL-2 levels may reflect RF titers to a certain extent. However, whether serum IL-2 levels change dynamically with RF titers and can be used as a reference for diagnosis and assessment of disease still needs to be demonstrated by further prospective longitudinal studies.

### 4.2. Serum IL-2 Levels Reflected Th17/Treg Immune Imbalance in RA Patients

Studies have shown that the balance between Th17 cells that promote the inflammatory response and Treg cells that maintain immune tolerance plays an important role in the disease progression of RA [17, 18]. Treg cells are a subgroup of CD4+ T lymphocytes that can suppress effector T cells and inflammation and play an important role in maintaining autoimmune tolerance [19]. Treg cells protect against arthritis in collagen-induced arthritis mouse models, in which depletion of Treg cells leads to exacerbation of arthritis; supplementation of exogenous Treg cells not only delays progression of the disease [20] but also inhibits generation of osteoclasts and promotes increased bone mass [21]. Many studies have shown that, compared with healthy individuals, the percentage of circulating Treg cells is reduced in RA patients [22], implying that the decrease in Treg cells is closely related to the disease progression of RA. Th17 cells, as proinflammatory cells, mediate pannus growth, osteoclast formation, and synovial neovascularization and are a key player in disease development [23].

In summary, an imbalance between Th17 and Treg cells is a key mechanism in the pathogenesis of RA. The influence of these lymphocyte subsets should be taken into account in the selection of clinical programs; the numbers and ratio of Th17 and Treg cells are important evaluation tools during the course of RA. This study confirmed Treg cell reduction and Th17/Treg immune imbalance in RA patients, especially in those with active RA. In addition, serum IL-2 levels were significantly correlated with Th17/Treg ratios, implying their significance in monitoring patients' Th17/Treg immune imbalance.

### 4.3. Compensatory Increase in Serum IL-2 Levels in Patients with Active RA

This study showed that the numbers of peripheral Treg cells were significantly higher in the remission group than in the low disease activity and moderate-high disease activity groups; that is, in patients with active RA, increased IL-2 levels paralleled a decrease in Treg cells. IL-2 was first discovered in 1976. It is a soluble factor that has the unique ability to promote the proliferation of T cells *in vitro*. Although it is produced mainly by activated CD4+ T cells, activated CD8+ T cells, NKT cells, and B cells can also produce smaller amounts of IL-2, and these cells secrete IL-2 through autocrine or paracrine methods [24].

In the past, IL-2 was considered a T cell growth factor, whose main function was to promote the proliferation and activation of effector T cells and perhaps also induce autoimmune responses. However, with the advent of IL-2 or IL-2R*α* (CD25) gene-deficient mice, this traditional view has been challenged; the main function of IL-2 in the body may be to maintain autoimmune tolerance rather than immune support and enhancement as traditionally considered. The latest research has demonstrated that IL-2 is a key cytokine that maintains the differentiation, proliferation, and function of Treg cells.

Selective expression of the IL-2 receptor is an important mechanism in its regulation of immune cell subsets. However, the receptor of IL-2 contains three subunits, IL-2R*α* (CD25), IL-2R*β* (CD122), and IL-2R*γ*c (CD132), and a heterodimer composed of IL-2R*β* (CD122) and IL-2R*γ*c (CD132) provides intermediate affinity receptors for IL-2, which are generally expressed in resting T cells, CD8+ memory T cells, and NK cells. However, the heterotrimer composed of these three subunits provides a high-affinity receptor for IL-2, which is transiently expressed in activated T cells, B cells, and NK cells, but is continuously expressed in Foxp3+ Treg cells, which are critical for the proliferation, maintenance, and function of Treg cells [25]. Amado et al. [26] found that IL-2 depletion did not affect the numbers of Treg cells in experimental mice but did cause an increase in IL-2 production by CD4+ T cells, implying that the ability of Treg cells to inhibit cytokine production by CD4+ T cells depends on serum IL-2 levels. In addition, depletion of Treg cells led to an increase in IL-2 levels in mice with type 1 diabetes, but the numbers of CD4+ T cells that secreted IL-2 did not increase significantly [27]. This indicates that IL-2 may regulate its own production by acting directly on CD4+ T cells (the main cells that produce IL-2) or by indirectly activating the inhibitory function of Treg cells. Therefore, when the number of Treg cells decreases, there is a compensatory increase in production of IL-2 by CD4+ T cells. However, how long this compensatory mechanism can be maintained still needs to be confirmed by further prospective longitudinal studies.

In addition to IL-2, our study showed that serum IL-4, IL-6, and INF-*γ* levels were significantly correlated with disease activity, and further multiple regression analysis showed that serum IL-2 and IL-6 levels were independent factors affecting disease activity. As a regulator of the Treg/Th17 balance, IL-6 can drive the polarization of CD4+ T cells to the Th17 phenotype and inhibit generation of Treg cells. TGF-*β* and IL-6 are the initiating factors for naive CD4+ T cells to differentiate into Th17 cells. Low-dose TGF-*β* is necessary for the production of human Th17 cells, and IL-6 secreted by activated DC cells is a key factor in the differentiation of naive CD4+ T cells into Th17 cells or Treg cells. In the absence of IL-6, TGF-*β* alone cannot induce naive CD4+ T cells to differentiate into Th17 cells but induces Foxp3 expression, allowing cells to acquire most of the Treg properties and thus maintaining the body's immune suppression [28]. Given the significant correlation between serum IL-6 levels and disease activity, we hypothesized that in patients with active RA, high levels of IL-6 drive Th17 cell generation and inhibit Treg cell production and peripheral Treg cell numbers decrease, increasing compensatory production of IL-2 by CD4+ T cells.

### 4.4. Serum IL-2 Levels in RA Patients Are Closely Related to NK Cells

NK cells are important immune cells in the body that are related to antitumor, antiviral infection, and immune regulation. They play a unique role in the human immune response and in the maintenance of immune homeostasis [29], the development and function of which are regulated mainly by external cytokines, among which IL-2 may play an important role in maintaining NK cell activity [30]. Studies have found that NK cells promote the progression of RA in the early stages of the disease; they can be detected in inflamed synovial tissue and constitute 20% of lymphocytes in synovial fluid [31]. On the one hand, NK cells can regulate the activity of B, T, macrophages, fibroblasts, and other cells by secreting cytokines such as TNF-*α* and INF-*γ*. On the other hand, synovial NK cells cultured *in vitro* can mediate the transformation of monocytes to osteoclasts, a process that may play an important role in the bone destruction associated with inflammatory arthritis [32]. Our study found that in patients with active RA, peripheral lymphocytes and CD4+ T cell subsets decreased to varying degrees, possibly due to lymphocyte depletion and the use of disease-modifying antirheumatic *drugs*. However, the numbers and percentages of NK cells increased significantly in the moderate-high disease activity group, and both were positively correlated with serum IL-2 levels. We speculate that high levels of IL-2 are an important predisposing factor in inducing the increase in NK cells in patients with active RA.

IL-2 is pleiotropic, and differences in its biological effects depend not only on different receptor compositions but also on differences in the thresholds of IL-2 signals received by cells. Signal thresholds are determined by the concentration of IL-2 in the environment, and the length of time the cell is exposed to the IL-2 signal [25]. When levels of IL-2 in the body are low, due to high expression of the IL-2R*α* subunit, the high-affinity receptors on Treg cells will preferentially bind to IL-2 and limit the acquisition of IL-2 by NK cells, thereby inhibiting the function of NK cells [27, 33], which indicates that Treg cells can restrict the function of NK cells by limiting acquisition of IL-2. However, with the increase in IL-2 levels and the lack of Treg cells, NK cells are induced to express IL-2R*β* and IL-2R*γ*c receptors, which respond to high concentrations of IL-2 and promote self-proliferation and cytotoxicity, thereby increasing self-response to target cells and rapidly responding to weak stimulation [33]. Another study showed that the restrictive effect of Treg cells on NK cells can be eliminated after human cytomegalovirus infection. That study found that there was a significant difference between NK cells with sufficient Treg and those in Treg-depleted mice after viral infection, and the Treg-mediated inhibition effect on NK cells may be exceeded by the immune effect of high levels of IL-2 during virus infection [34]. Moreover, the NK cell proliferation promoted by high levels of IL-2 will further inhibit the proliferation of Treg cells. Studies by Heon et al. [35] have shown that in the presence of IL-2, NK cells inhibit the growth and development of Treg cells by secreting INF-*γ*. Our study also found that decreased Treg numbers in patients with active RA paralleled increased serum IL-2 levels and peripheral blood NK cell counts, and serum INF-*γ* levels were also correlated with the percentage of NK cells. This implies that when Treg cells decrease, high levels of IL-2 promote proliferation of NK cells, which may inhibit the function of Treg cells by secreting INF-*γ*.

We hypothesized that peripheral Treg cell numbers decrease in patients with active RA, resulting in a compensatory increase in serum IL-2 levels. High levels of IL-2 may promote NK cell proliferation and secretion of INF-*γ*, inhibiting proliferation of Treg cells and aggravating the Th17/Treg immune imbalance, thereby promoting the disease progression of RA. IL-2 has been used in clinical practice for more than 30 years. High doses of IL-2 are often used to treat malignant tumors, because IL-2 preferentially induces activation and proliferation of effector T cells (Teff cells), NK cells, and other cells; however, the effectiveness of this treatment is limited by toxicity and instability [36]. In recent years, many studies have shown that Treg cells continue to express high levels of IL-2R*α*, and low-dose IL-2 preferentially promotes activation and proliferation of Treg cells [37]; therefore, exogenous administration of low doses of IL-2 can selectively stimulate Treg cell proliferation without triggering effector T cell proliferation. This concept has been widely used in clinical practice, and currently, low-dose IL-2 is being used for the treatment of diseases, including graft-versus-host disease (GVHD), TID, hepatitis C virus-induced vasculitis, spinal arthropathy, and SLE [24, 38]. In addition, low-dose IL-2 reportedly promotes the proliferation and differentiation of Treg cells affected by chronic activation in patients with invasive RA, supporting its use in progressive RA therapy [39]. Our research also found that low levels of IL-2 are effective signals for Treg cell activation and proliferation; therefore, low-dose IL-2 can be used to stimulate Treg cell proliferation and restore immune tolerance during treatment of autoimmune diseases such as RA. However, large doses of IL-2 may inhibit the function of Treg cells by stimulating NK cell proliferation.

Our study had several limitations. First, it included patients from the same medical center, and the clinical and serological parameters were collected retrospectively. Second, serum IL-2 levels in RA patients were affected by many confounding factors, such as the choice of treatment. Finally, the molecular biological mechanism of IL-2, NK cells, and the Th17/Treg immune imbalance in RA patient needs further study. We will conduct a prospective cohort longitudinal study of RA patients and further expand the sample size, observe whether IL-2 levels increase in all individuals, determine whether the increase in IL-2 is indeed related to a reduction in Treg cells and proliferation and excessive secretion of NK cells, and carry out basic research to explore the molecular biological mechanisms further.

To summarize, monitoring serum IL-2 levels has important clinical significance in RA patients, because IL-2 levels not only reflect the degree of inflammation, disease activity, arthralgia, and the production of autoantibodies but also reflect the immune imbalance of Th17/Treg in patients. More importantly, in patients with active RA, NK cells are abnormally elevated, possibly due to high serum IL-2 levels.

## Figures and Tables

**(a):** 

	Remission group (A) (*n* = 33)	Low disease activity group (B) (*n* = 38)	Moderate-high disease activity group (C) (*n* = 35)	*p* A *vs*. B	*p* A vs. C	*p* B vs. C
Demographics						
Age (years)^a^	45.09 ± 13.77	47.18 ± 16.41	49.20 ± 14.60	0.474	0.302	0.778
Male, *n* (%)	11 (33.33%)	4 (10.53%)	10 (28.57%)	—	—	—
Female, *n* (%)	22 (66.67%)	34(89.47%)	25 (71.43%)	—	—	—
Course of disease (month)^b^	10.00 6.25-12.75	9.00 5.75-15.25	12.00 7.00-17.00	0.957	0.273	0.342
Height (m)^a^	1.63 ± 0.07	1.61 ± 0.063	1.63 ± 0.072	0.632	0.861	0.502
Weight (kg)^a^	64.39 ± 16.96	58.95 ± 9.92	59.69 ± 10.15	0.286	0.740	0.635
BMI^a^	24.10 ± 4.60	22.66 ± 3.22	22.34 ± 3.16	0.256	0.175	0.781
ESR, (mm/h)^b^	10.50 6.00-15.75	20.00 10.00-34.25	43.00 33.00-63.00	0.003	*<* 0.001	*<* 0.001
CRP (mg/ml)^b^	4.10 2.56-7.41	7.273.66-16.30	10.104.88-22.48	0.221	0.008	0.134
VAS score^b^	10.00 5.00-15.00	15.00 11.25-25.00	35.00 25.00-50.00	0.002	*<* 0.001	0.001
DAS28 score ^a^	1.87 ± 0.60	2.78 ± 0.39	4.84 ± 0.67	*<* 0.001	*<* 0.001	*<* 0.001
WBC (^∗^10^9^/l)^b^	6.80 5.30-8.77	6.80 5.45-9.05	7.40 5.00-9.10	0.782	1.000	0.710
Hb (g/l)^a^	137.95 ± 16.77	124.75 ± 13.56	123.11 ± 16.36	0.001	*<* 0.001	0.527
PLT (^∗^10^9^/l)^b^	229.00 187.50-277.40	237.50 204.00-308.60	251.00 179.00-315.00	0.340	0.404	0.968
LY (^∗^10^9^/l)^b^	2.10 1.60-2.47	1.79 1.31-2.50	1.68 1.05-2.19	0.058	0.009	0.446
RF (U/ml)^b^	82.35 40.00-184.75	80.00 40.00-294.80	80.00 40.00-160.00	0.484	0.835	0.362
Anti-CCP (RU/ml)^b^	397.45 39.98-1476.66	598.85 130.76-1300.03	394.80 187.90-1000.00	0.659	0.569	0.771

**(b):** 

	Remission group (A) (*n* = 33)	Low disease activity group (B) (*n* = 38)	Moderate-high disease activity group (C) (*n* = 35)	*p*, A *vs*. B	*p*, A vs. C	*p*, B vs. C
Medication type						
NSAIDS, *n* (%)	11 (33.33%)	27 (71.05%)	22 (62.86%)	—	—	—
CS, *n* (%)	12 (36.36%)	26 (68.42%)	25 (71.43%)	—	—	—
Oral prednisone dose (mg/day)^b^	7.50 4.38-10.00	10.00 5.00-12.50	10.00 8.75-13.75	0.139	0.015	0.311
Prednisone use time (month)^b^	3.65 2.80-4.00	4.00 2.75-5.25	4.00 2.25-5.00	0.076	0.546	0.301
MTX, *n* (%)	3 (9.09%)	5 (13.16%)	5 (14.29%)	—	—	—
LEF, *n* (%)	3 (9.09%)	3 (7.89%)	4 (11.43%)	—	—	—
HCQ, *n* (%)	5 (15.16%)	7 (18.42%)	5 (14.29%)	—	—	—
TNF-ɑ inhibitors, *n* (%) (50 mg/week)	5 (15.16%)	9 (23.68%)	12 (34.29%)	—	—	—
TNF-ɑ inhibitor use time (week)^b^	5.50 2.50-7.75	6.00 3.00-11.00	7.00 3.00-9.00	0.624	0.489	0.691

^a^Results are expressed as the mean ± standard deviation. ^b^Results are expressed as the median and 25th and 75th percentiles. The independent-samples *t*-test was used for analysis of quantitative variables with normal distributions. Wilcoxon's rank sum test was used for analysis of quantitative variables with a nonnormal distribution. Abbreviations: RA: rheumatoid arthritis; BMI: body mass index; ESR: erythrocyte sedimentation rate; CRP: C-reaction protein; VAS: visual analogue scale; WBC: white blood cells; Hb: hemoglobin; PLT: platelets; LY: lymphocytes; RF: rheumatoid factor; Anti-CCP: anticyclic citrullinated peptide antibodies; MTX: methotrexate; LEF: leflunomide; HCQ: hydroxychloroquine; CS: corticosteroid; NSAID: nonsteroidal anti-inflammatory drug.

**Table 2:** Serum cytokine levels of RA patients in each disease activity group.

Cytokine (pg/ml)	Remission group (A) (*n* = 33)	Low disease activity group (B) (*n* = 38)	Moderate-high disease activity group (C) (*n* = 35)	*p*, A vs. B	*p*, A vs. C	*p*, B vs. C
IL-2	3.39 2.49-6.25	3.71 1.67-5.29	8.03 3.52-18.07	0.893	0.002	0.001
IL-4	2.92 1.16-3.89	2.13 1.52-4.22	4.23 2.05-7.88	0.991	0.044	0.029
IL-6	13.05 6.53-31.07	16.12 7.22-46.40	47.85 22.20-109.12	0.473	*<*0.001	0.001
IL-10	5.83 2.95-8.41	4.67 3.07-7.91	9.28 3.33-15.20	0.600	0.068	0.024
INF-*γ*	6.45 3.69-9.75	4.39 2.20-10.52	13.11 5.70-23.32	0.460	0.006	*<*0.001
TNF-*α*	5.93 2.57-14.69	2.78 1.90-8.05	7.78 3.88-15.58	0.067	0.367	0.005

Results are expressed as the median and 25th and 75th percentiles. Statistics: Wilcoxon's rank sum test. Abbreviations: IL-2: interleukin-2; IL-4: interleukin-4; IL-6: interleukin-6; IL-10: interleukin-10; INF-*γ*: interferon-*γ*; TNF-*α*: tumor necrosis factor-*α*.

**Table 3:** Multiple linear regression analysis of DAS28 score and cytokines in patients with RA.

	B	SE	*β*	*t*	*p*
Constant	2.874	0.162		17.724	*<*0.001
IL-2	0.020	0.010	0.237	2.059	0.042
IL-4	-0.003	0.020	-0.107	-0.103	0.897
IL-6	0.002	0.001	0.196	2.044	0.044
INF-*γ*	0.009	0.007	0.154	1.357	0.178

Statistics: Multiple linear regression analysis. Abbreviations: IL-2: interleukin-2; IL-4: interleukin-4; IL-6: interleukin-6; IL-10: interleukin-10; INF-*γ*: interferon-*γ*; TNF-*α*: tumor necrosis factor-*α*.

**(a):** 

	Total T	Total T%	Total B	Total B%
Remission group (A) (*n* = 33)	1664.60 1296.80-2078.74	74.88 66.89-76.95	237.98 176.73-393.59	11.00 8.56-14.13
Low disease activity group (B) (*n* = 38)	1293.00 914.85-1707.04	73.89 65.45-78.00	178.11 136.50-282.50	11.00 8.63-14.12
Moderate-high disease activity group (C) (*n* = 35)	1223.00 651.00-1698.00	72.16 68.72-76.77	159.60 103.36-273.15	10.01 7.83-15.00
*p*, A vs. B	0.017	0.986	0.029	0.940
*p*, A vs. C	0.003	0.816	0.001	0.615
*p*, B vs. C	0.493	0.761	0.281	0.683

**(b):** 

	NK	NK%	CD4+	CD8+	Th1
Remission group (A) (*n* = 33)	237.00 139.00-301.00	12.51 7.50-15.99	848.64 671.28-1182.02	851.87 456.51-876.82	158.23 110.04-267.17
Low disease activity group (B) (*n* = 38)	218.50 144.50-299.75	12.20 7.60-18.75	633.74 497.18-953.50	472.17 304.34-713.09	119.72 62.96-199.36
Moderate-high disease activity group (C) (*n* = 35)	283.00 217.00-370.00	18.46 15.26-23.47	765.80 434.00-981.00	373.97 214.12-505.40	96.92 68.97-184.99
*p*, A vs. B	0.808	0.669	0.018	0.026	0.018
*p*, A vs. C	0.046	<0.001	0.118	<0.001	0.011
*p*, B vs. C	0.020	0.001	0.817	0.090	0.812

**(c):** 

	Th2	Th17	Treg	Th1/Th2	Th17/Treg
Remission group (A) (*n* = 33)	7.50 4.86-11.17	11.31 7.41-14.56	34.76 27.43-44.60	26.58 18.02-39.57	0.29 0.23-0.34
Low disease activity group (B) (*n* = 38)	5.34 3.41-8.40	7.52 4.79-10.92	27.92 16.34-38.28	20.56 14.92-28.54	0.29 0.20-0.44
Moderate-high disease activity group (C) (*n* = 35)	5.51 3.41-8.72	7.71 5.23-13.59	25.12 13.06-30.58	18.86 14.50-28.30	0.40 0.28-0.62
*p*, A vs. B	0.042	0.012	0.005	0.050	0.668
*p*, A vs. C	0.099	0.097	<0.001	0.031	0.003
*p*, B vs. C	0.982	0.406	0.108	0.707	0.010

Results are expressed as the median and 25th and 75th percentiles. Statistics: Wilcoxon's rank sum test. Abbreviations: RA: rheumatoid arthritis; Total T: total T cells; Total T%: T lymphocyte percentage; Total B: total B cells; Total B%: B lymphocyte percentage; NK: natural killer T cells; NK%: percentage of NK cells; CD4+: CD4+ T cells; CD8+: CD8+ T cells; Th1: T helper 1 cells; Th2: T helper 2 cells; Th17: T helper 17 cells; Treg: regulatory T cells; Th1/Th2: T helper 1 cell/T helper 2 cell ratio; Th17/Treg: T helper 17 cell/regulatory T cell ratio.

**Table 5:** Relationship between serum IL-2 level and peripheral blood lymphocytes and CD4+ T cells.

	*r*	*p*
IL-2 vs. Total T	-0.084	0.392
IL-2 vs. Total T%	-0.111	0.259
IL-2 vs. Total B	-0.097	0.320
IL-2 vs. Total B%	-0.087	0.378
IL-2 vs. NK	0.230	0.018
IL-2 vs. NK%	0.268	0.006
IL-2 vs. CD4+	0.032	0.745
IL-2 vs. CD8+	-0.022	0.825
IL-2 vs. Th1	-0.024	0.804
IL-2 vs. Th2	0.108	0.270
IL-2 vs. Th17	0.153	0.117
IL-2 vs. Treg	-0.026	0.789
IL-2 vs. Th1/Th2	-0.149	0.128
IL-2 vs. Th17/Treg	0.239	0.013

Statistics: Spearman's correlation tests. Abbreviations: RA: rheumatoid arthritis; Total T: total T cells; Total T%: T lymphocyte percentage; Total B: total B cells; Total B%: B lymphocyte percentage; NK: natural killer T cells; NK%: percentage of NK cells; CD4+: CD4+ T cells; CD8+: CD8+ T cells; Th1: T helper 1 cells; Th2: T helper 2 cells; Th17: T helper 17 cells; Treg: regulatory T cells; Th1/Th2: T helper 1 cell/T helper 2 cell ratio; Th17/Treg: T helper 17 cell/regulatory T cell ratio.

## Data Availability

The Research Article data used to support the findings of this study are available from the corresponding author upon request.
